# A Bayesian Model of the Memory Colour Effect

**DOI:** 10.1177/2041669518771715

**Published:** 2018-05-07

**Authors:** Christoph Witzel, Maria Olkkonen, Karl R. Gegenfurtner

**Affiliations:** Universität Gießen, Allgemeine Psychologie, Gießen, Germany; Department of Psychology, Durham University, Durham, UK; Department of Psychology and Logopedics, Faculty of Medicine, University of Helsinki, Finland; Universität Gießen, Allgemeine Psychologie, Gießen, Germany

**Keywords:** bayesian model, cognitive penetration, colour vision, memory, object recognition

## Abstract

According to the memory colour effect, the colour of a colour-diagnostic object is not perceived independently of the object itself. Instead, it has been shown through an achromatic adjustment method that colour-diagnostic objects still appear slightly in their typical colour, even when they are colourimetrically grey. Bayesian models provide a promising approach to capture the effect of prior knowledge on colour perception and to link these effects to more general effects of cue integration. Here, we model memory colour effects using prior knowledge about typical colours as priors for the grey adjustments in a Bayesian model. This simple model does not involve any fitting of free parameters. The Bayesian model roughly captured the magnitude of the measured memory colour effect for photographs of objects. To some extent, the model predicted observed differences in memory colour effects across objects. The model could not account for the differences in memory colour effects across different levels of realism in the object images. The Bayesian model provides a particularly simple account of memory colour effects, capturing some of the multiple sources of variation of these effects.

## Introduction

How does memory influence perception? The memory colour effect shows that knowledge about the typical colour of an object affects how we perceive the actual colour of that object (Olkkonen, Hansen, & Gegenfurtner, 2012; Witzel & Gegenfurtner, 2014; Witzel & Hansen, 2015). For example, a banana is perceived as slightly yellow even when it is colourimetrically grey (Hansen, Olkkonen, Walter, & Gegenfurtner, 2006).

Such memory colour effects have been shown in an achromatic adjustment task, where observers are asked to adjust images of objects so that the objects look grey to them. In this task, observers perceive objects as slightly tinted in their typical colour when they are colourimetrically grey. As a consequence, they adjust the objects approximately to the opponent colour in order to compensate for the typical colour. For example, bananas are adjusted slightly towards the blue direction in order to perceive them as grey. This has been shown for different objects with different typical colours (Hansen et al., 2006; Kimura et al., 2013; Olkkonen, Hansen, & Gegenfurtner, 2008; Witzel, Valkova, Hansen, & Gegenfurtner, 2011). Observers also tend to choose a bluish banana over a colourimetrically grey banana when they are asked to choose the one that looks most grey (Witzel, 2016).

A recent article contested the idea of top-down influences on perception and suggested that memory colour effects might not involve visual perception at all (Firestone & Scholl, 2016). According to Firestone and Scholl (2016), perception can be clearly separated from cognition, and there is no role for cognitive factors such as prior knowledge and memory in perceptual processing. This is shown, they argue, by the fact that common models in vision science do not involve such cognitive factors (Firestone & Scholl, 2016, pp. 1–2).

However, theoretically, a simple Bayesian model might well incorporate memory colour effects (Witzel, Olkkonen, & Gegenfurtner, 2016). According to the memory colour effect, the perception of colour-diagnostic objects is a combination of the sensory signal of perceived greyness and the expectation based on memory colours. This idea is captured by a simple Bayesian model that integrates the sensory signal of perceived greyness and the prior about memory colours to predict the empirically measured memory colour effects.

Bayesian models have been used successfully to capture the effects of prior knowledge on perception, for instance in colour constancy (Brainard et al., 2006), orientation perception (Girshick, Landy, & Simoncelli, 2011) and temporal interval estimation (Jazayeri & Shadlen, 2010), as well as perceptual cue integration (Ernst & Banks, 2002). A recent study also modelled effects of linguistic colour categories on short-term colour memory (Cibelli, Xu, Austerweil, Griffiths, & Regier, 2016).

Applying the Bayesian framework on the memory colour effect, the observer’s perceptual estimate is calculated by combining the noisy sensory signal inherent in the perception of grey colour with knowledge about the typical colour of an object, the *prior*. According to Bayes’ rule, the final perceptual estimate (the *posterior*) depends on the reliabilities of the sensory signal and of the prior, with more shift or bias towards the prior when uncertainty in the sensory signal increases (Knill & Richards, 1996; Maloney & Mamassian, 2009).

Such a Bayesian model is particularly simple: It only requires two measured variables (prior and sensory signal) to predict the dependent variable (memory colour effects). Hence, it is completely determined by empirical data and does not require any fitting of parameters. A Bayesian model of memory colour effects links these effects to more general cue-integration effects where different sources of information are combined (Witzel & Gegenfurtner, in press). This would create a comprehensive framework for understanding these kinds of effects.

A Bayesian model seems particularly promising for at least two reasons. First, Witzel et al. (2011) observed a correlation between memory colour effects and colour diagnosticity for the stimuli of the first experiment of Olkkonen et al. (2008). Colour diagnosticity was measured through response times in a two-alternative forced choice (2AFC) task, in which observers had to report the typical colour of greyscale objects by keypress. Per definition, colour diagnosticity is based on knowledge about the typical colour (Witzel & Gegenfurtner, 2014; Witzel & Hansen, 2015). Consequently, our definition of the prior is conceptually related to the response time measure of colour diagnosticity; although the prior used here is determined differently from that measure of colour diagnosticity (mean and reliability of typical adjustments instead of response times in a different task). At the same time, the response time measure of colour diagnosticity explained only a relatively small part of the variance (about 27%), and it only occurred for the stimuli of Olkkonen et al. (2008) but not for those of Witzel et al. (2011). These limitations suggested that factors or models other than simple regressions might provide a better account of memory colour effects (cf. discussion in Witzel et al., 2011).

Second, one other factor might be the uncertainty of the sensory signal, that is, the grey adjustments. According to previous observations, memory colour effects are related to the uncertainty of grey perception along the blue–yellow direction of the daylight locus (Witzel & Hansen, 2015; Witzel et al., 2011). The daylight locus is a curve in colour space along which natural daylight varies (Granzier & Valsecchi, 2014; Judd et al., 1964; Mollon, 2006; Taylor & Kerr, 1941). Variation of grey adjustments is largest on the daylight locus, indicating that observers are uncertain about colour appearance along this colour direction (Bosten, Beer, & MacLeod, 2015; Chauhan et al., 2014; Witzel, Racey, & O’Regan, 2017; Witzel et al., 2011; Wuerger, Hurlbert, & Witzel, 2015). At the same time, memory colour effects were larger for objects whose typical colours are closer to the daylight locus (Witzel & Hansen, 2015; Witzel et al., 2011). The Bayesian model would explain why the uncertainty of grey settings along the daylight locus modulates the magnitude of memory colour effects: The higher the uncertainty of the signal (grey settings), the higher the influence of the prior (memory colours).

In this study, we empirically tested the idea that a standard Bayesian model may predict the magnitude of memory colour effects. The strength of associations between objects and their typical colours depends on the observers’ knowledge and the objects themselves (Witzel et al., 2011). Hence, different observers and different objects imply different priors. If the Bayesian approach applies in the case of memory colour, a Bayesian model should predict variation in the magnitude of the memory colour effects across different objects and observers. We tested these predictions with the data from the studies of Hansen et al. (2006), Olkkonen et al. (2008), and Witzel et al. (2011).

## General Method

### Stimuli

All images of objects were presented on a grey background. The luminance of the images was adjusted to be on average isoluminant with the background. Colours were represented in the Derrington–Krauskopf–Lennie colour space (Derrington, Krauskopf, & Lennie, 1984; Krauskopf, Williams, & Heeley, 1982), for which the origin corresponded to the grey of the background.

### Procedure

**Figure 1.**
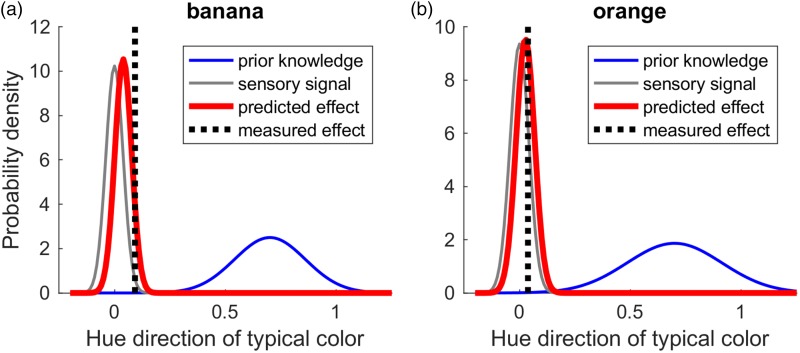
Illustration of the Bayesian model of the memory colour effect. The model predictions and data shown here are based on the aggregated data (cf. ‘third approach to test the models’) taken from Hansen et al. (2006). (a and b) Illustration of a model for the data of the banana and the orange, respectively. The *x*-axis corresponds to the adjustments projected onto the hue direction of the typical colour of the respective object. In the case of the banana (a) the adjustments vary along a blue–yellow dimension, in the case of the orange along a turquoise-orange dimension. The *y*-axis shows the probability density of adjustments along a respective hue direction (*x*-axis). The thin grey and the blue curves show normal distributions fitted to the empirical adjustments of the grey disk (sensory signal) and of the typical colour of the object (prior knowledge), respectively. The vertical dotted line corresponds to the average memory colour effect resulting from the grey adjustments of the objects. The thick red curve shows the memory effect predicted by the Bayesian model. The proximity between the peak of the red curve and the dotted black line indicates how well the model predicted the empirically measured effect.

In all experiments, three types of empirical measurements were obtained:

1. **Typical adjustments of colour-diagnostic objects:** Observers adjusted the colour of the image of the object so that it had the typical colour of the object, as remembered by the observer. The knowledge about the typical colour defines the expected colour of the object. The average and the standard deviation of these adjustments were used to model the *prior* (blue curve in Figure 1). In other words, the precision of the colour in the observer’s memory defines the strength of the expected colour of a particular object.
2. **Achromatic adjustments of a disk:** Observers adjusted the colour of a colour-neutral disk so that it looked grey to them. The disk does not have a memory colour, and hence its adjustment only varies because of noise in the perception of grey (Plus some motor noise, which we disregarded here). The average and the standard deviation of these adjustments were used to model the *sensory signal* (grey curve in Figure 1). In other words, the standard deviation defined the certainty of the sensory signal when perceiving a grey colour.
3. **Grey adjustments of colour-diagnostic objects:** Observers adjusted the colour of the image of colour-diagnostic objects so that they looked grey to them. Our aim is to predict the average of these measures (dotted vertical line in Figure 1) through the average of the *posterior* calculated based on prior and sensory signal in the Bayesian model (red curve in Figure 1).

To adjust the colours, observers could press four keys to add red, yellow, green and blue. Adjustments were translated into polar coordinates, resulting in a hue shift and a rescaling of saturation of the colour distribution. Measures of colour adjustments report the coordinates of the most saturated colour of the colour distribution. Details on the achromatic adjustment procedure may be found in our previous publications (Hansen et al., 2006; Olkkonen et al., 2008; Witzel & Hansen, 2015; Witzel et al., 2011, 2016).

### Bayesian Model

Memory colour effects are quantified along the chromatic dimension of the memory colour, for example, along the blue–yellow dimension of the typical yellow of the banana (Hansen et al., 2006; Olkkonen et al., 2008; Witzel & Hansen, 2015; Witzel et al., 2011, 2016). Accordingly, we assessed uncertainty along the chromatic dimension of the memory colour. Typical and grey adjustments were projected onto that dimension, and averages and standard deviations were calculated for the projected coordinates. Projected coordinates of grey adjustments provided the uncertainty of the signal, those for the typical adjustments the uncertainty of the prior.

The average (*M*) of the predicted memory colour effects (posterior, red curve in Figure 1) was calculated based on the weighted average of sensory signal (grey curve in Figure 1) and the prior (blue curve in Figure 1), where the weights correspond to their relative reliability (Ernst, 2006; Ernst & Banks, 2002). The weighted average is calculated as follows:

M=w1×P+w2×S

where *P* is the average of the prior and *S* is the average of the signal; *w*1 and *w*2 are the weights, which are calculated as follows:

$$w1=\frac{r1}{r1+r2}$$

where *r*1 and *r*2 are the reliabilities of the prior and the signal, respectively. Reliabilities were calculated as (Backus & Banks, 1999):

$$r=\frac{1}{\mathrm{var}}$$

Finally, the standard deviation of the posterior (*SD*) was calculated as follows:

$$\mathrm{SD}=\sqrt{\frac{1}{(r1+r2)}}$$

We tested the relationship between measured memory colour effects and the predictions from a Bayesian model in three ways. First, we computed the Bayesian model for each individual observer. In all experiments, each observer completed three to five repeated measurements of grey adjustments and typical adjustments (e.g., the memorized yellow of the banana). Observers also made grey adjustments with disks, which are colour-neural. The variance across repeated measurements of the disk’s grey adjustments was used to calculate the reliability of the signal. Similarly, the variance for the memory colour adjustments for each object was used to calculate the reliabilities of the respective priors.

In the first approach to test the model, we correlated the predicted memory colour effect with the measured memory colour effect for each individual separately. Then we Fisher transformed the individual correlation coefficients and tested with a *t* test across individuals whether the correlation was positive.

In the second approach to test the model, we pooled all the data across all individuals and all stimuli in one dataset. Then, we calculated a correlation between the predicted and measured effects. In this approach, the variation across individual observers is also part of the variation in this dataset. This approach also provides a particularly high statistical power.

Finally, the third approach was based on the aggregated data. The calculation of the Bayesian model requires specifying variances, but the variance of average data differs from the variance of individual data (cf. the difference between standard deviation and standard error). The averages of each observer are normally distributed and the variance of those averages corresponds to the variance across repeated measurements divided by the number of repeated measurements (var/n). Hence, we used the variance of averages to calculate the reliability of signals and priors for the aggregate data.

## Study of Hansen et al. (2006)

### Method

Fourteen observers participated in the experiment of Hansen et al. (2006). The colour diagnostic objects were seven fruits and vegetables: a banana, lemon, carrot, orange, lettuce, grapes and courgette. In all experiments, there were two kinds of disks, one with uniform colour and one with a luminance noise texture; adjustments of these disks are pooled and presented as one measurement. Observers completed five typical and five grey adjustments for each colour-diagnostic object and five grey adjustments of the colour-neutral disk.

### Results and Discussion

**Figure 2.**
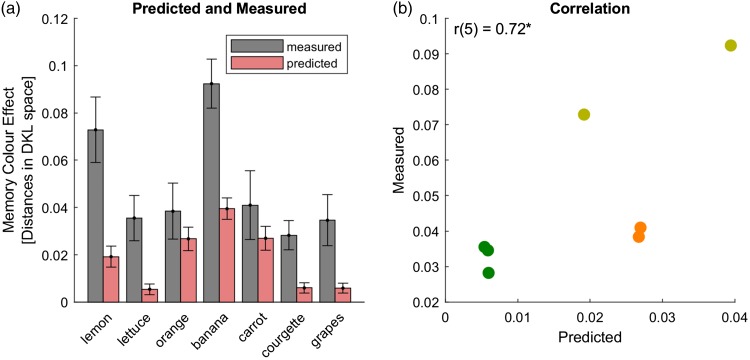
Predictions for aggregated data of Hansen et al. (2006). (a) Measured (grey) and predicted (red) memory colour effects. The *x*-axis lists the seven objects and the *y*-axis represents the shifts along the hue direction of the typical colour. Error bars correspond to standard errors of mean, across individual measurements and based on the standard deviation of the model, respectively. (b) Correlation between predicted (*x*-axis) and measured (*y*-axis) memory colour effects. The correlation is reported in the upper left corner. *p < .05 in a one-tailed *t* test.

Figure 1 illustrates the predictions of the model using the aggregated data for the banana and orange. In general, the model predictions (shown with the red curves) were similar in magnitude to the measured effects (vertical dotted lines), although the model sometimes underestimated the magnitude of the effect. This is illustrated by Figure 2(a), which shows predicted (red bars) and measured (grey bars) memory colour effects side by side. Measured memory colour effects were significantly larger than the ones predicted by the Bayesian model, Δ_pred-meas_ = 0.03, *t*(6) = 4.7, *p* = .003. However, predicted effects were positively correlated with the measured ones in a one-tailed *t* test, *r*(5) = .72, *p* = .03 (Figure 2(b)). This correlation supports the idea that our Bayesian model explains the variation of memory colour effects across stimuli.

## Study of Olkkonen et al. (2008)

The study of Olkkonen et al. (2008) reports two experiments. The data from the first experiment provide an interesting test case. That experiment used images with different levels of perceptual features and recognizability. We could show that the magnitude of the memory colour effect decreased with image information and recognizability (Figure 4 in Olkkonen et al., 2008; Figure 1 in Witzel et al., 2011). However, our Bayesian model is only designed to model the influence of knowledge about typical colours but does not model image information and recognizability. If the observed relationship between predicted and measured memory colours is specific to knowledge about typical colours, the model should fail to predict the variation of the effect across different levels of perceptual features and recognizability. At the same time, the model should predict the variation of memory colour effects across objects within a given level of perceptual features and recognizability.

The second of the two experiments measured memory colour effects under changing illuminations. The data in that experiment showed higher variation, which is probably due to variation in colour constancy across colours and objects. We did not consider modelling the data from the second experiment because our Bayesian model is not appropriate for variation of colour constancy and must fail to predict the measured effects.

The first experiment measured memory colour effects under neutral illumination with different sets of images in two subexperiments. To simplify, we will distinguish these two subexperiments as Experiments 1a and 1b.

### Method

#### Experiment 1a

Fifteen observers took part in Experiment 1a. The stimulus images were photos and outline shapes of eight fruits and vegetables. Seven of these corresponded to the fruits and vegetables used by Hansen et al. (2006). The eighth was a photo and outline shape of strawberry. As in Hansen et al. (2006), observers completed five repeated measurements in Experiments 1a and 1b.

#### Experiment 1b

Seven observers participated in Experiment 1b. The stimulus images in Experiment 1b featured five of the eight fruits and vegetables from Experiment 1a, namely, banana, courgette, grapes, lemon and strawberry. Besides the photos and outline shapes, this experiment also included photos of objects that were painted in white so that they did not have their natural texture.

### Results and Discussion

#### Experiment 1a

**Figure 3.**
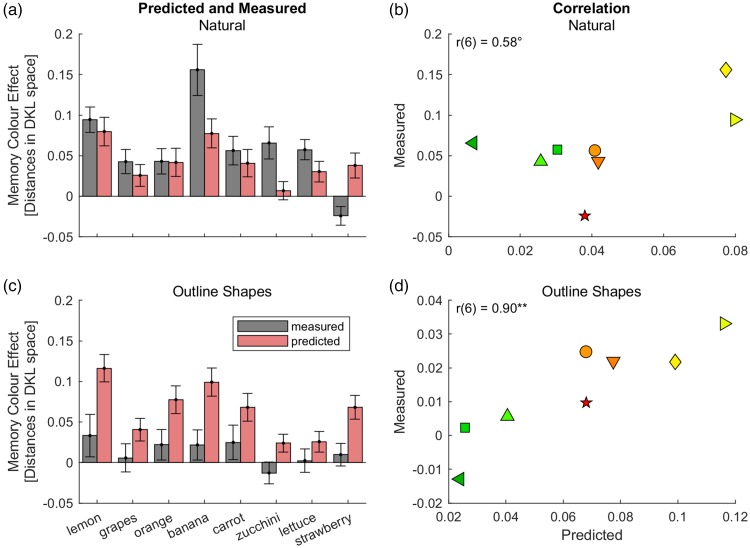
Predictions for aggregated data of Experiment 1a of Olkkonen et al. (2008). The upper row ((a) and (b)) illustrates results for photographs, the lower row ((c) and (d)) those for the outline shapes. The grey bars correspond to the bars in Figure 4(a) of Olkkonen et al. (2008). Note, however, that the bars in this figure show absolute shifts of grey adjustments in the opposite direction of typical adjustments, the Memory colour Indices shown in Olkkonen et al. (2008) represent relative shifts, that is, the absolute shifts relative to the typical adjustments. **p < .01; °*p* < .1.

Figure 3(a) and (c) illustrates predicted and measured memory colour effects for aggregated data in Experiment 1a. For the original photographs (Figure 3(a)), predicted memory colour effects were similar in size to measured memory colour effects, Δ_pred-meas_ = − 0.019, *t*(7) = −1.3, *p* = .24. In contrast, measured memory colour effects were significantly smaller than predicted effects for the outline shapes, Δ_pred-meas_ = 0.05, *t*(7) = 7.0, *p* = .0002; cf. (Figure 3(c)). This reflects the fact that measured memory colour effects were much smaller for outline shapes than for photographs (compare grey bars in Figure 3(c) and Figure 3(a)), while predicted effects were comparable across conditions (red bars in Figure 3(a) and (c)).

**Figure 4.**
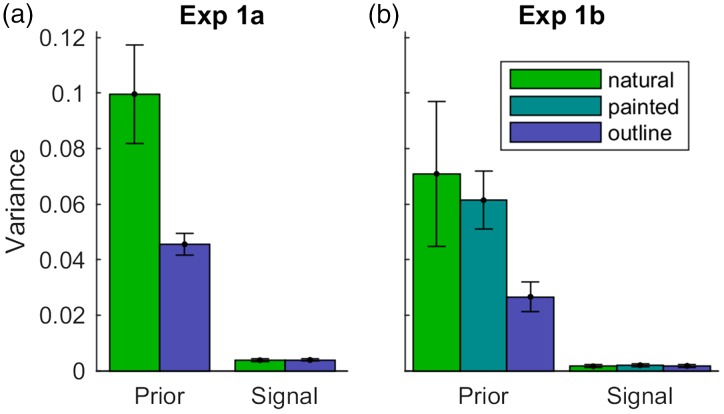
Comparison of variance in colour adjustments across conditions. (a and b) Experiments 1a and 1b of Olkkonen et al. (2008). The different bar colours refer to the different conditions, that is, those with photographs (green), with outline shapes (blue) and with painted fruits (green–blue). The *y*-axis represents the variance of adjustments for the prior (typical adjustments) and the signal (grey adjustments) averaged across observers and stimuli. To give an idea of variation, error bars indicate standard errors of mean across the eight stimuli. The variances inform about the reliability of adjustments. Note that variance for typical adjustments was higher (i.e., lower reliability) for photographs of natural and painted fruits than for uniformly coloured outline shapes.

As expected, our Bayesian model cannot explain the differences between natural images and outline shapes. The difference between the two conditions (photograph vs. outline shape) is not a difference in certainty about memory colours but a difference of perceptual features and recognizability of the outline shapes (Olkkonen et al., 2008; Witzel et al., 2011). The uncertainty about the memory colour is the same for both conditions because the association between the object and its colour in the observer’s memory does not change. Hence, predicted memory colour effects based on our Bayesian models are the same. For this reason, it is understandable that our Bayesian models cannot explain the difference across these conditions.

This idea is further supported by a closer look at the reliability of the adjustments (Figure 4(a)). If the smaller memory colour effects for the outline shapes were related to the reliability of the object–colour association captured by our Bayesian model, then the reliability of typical adjustments should be lower (i.e., variance higher) in the condition with the outline shapes than in the condition with the photographs. The contrary was true: Variance of typical adjustments was higher for natural photographs (green bars) than for outline shapes (blue bars), indicating that adjustments for outline shapes were more reliable than those for photographs. Since grey adjustments are similarly reliable in both conditions, the Bayesian model provides predictions of slightly higher memory colour effects for outline shapes than for photographs, which contradicts the differences in measured memory colour effects.

With respect to the variation across stimuli, there was a positive correlation between predicted and measured memory colour effects for outline shapes, *r*(6) = .90, *p* = .001; Figure 3(b). The correlation coefficient for the photographs was also positive, but only marginally significant, *r*(6) = 0.58, *p* = .07, one-tailed, Figure 3(a). Combining the data from both conditions (natural and outline shapes) did not yield significant results, *r*(14) = .20, *p* = .46, one-tailed. This is understandable given that the Bayesian model cannot account for the difference across conditions. To account for the difference between the two conditions we calculated a ‘relative memory colour effect’, which consists in the difference between the memory colour effect for each stimulus and the average memory colour effect for the condition (natural vs. outline). The predictions from the model are positively correlated with the variation of these relative memory colour effects across the 2 × 8 stimuli, *r*(14) = .50, *p* = .03, one-tailed.

We then tested the correlation between predicted and measured effects across individual observers (first approach to test the model). To combine both conditions (photographs and outline shapes), we averaged for each observer and each object Fisher-transformed correlation coefficients across conditions. We calculated the correlation between predicted and measured effects for each observer. Then we tested with a *t* test whether the Fisher-transformed correlation coefficients were above zero. The average resulting correlation coefficient was significantly positive, average *r* = .16, *t*(14) = 2.0, *p* = .03, one-tailed. Together with the result for aggregated data, these findings support the idea that the Bayesian model predicts, to some extent, the measured memory colour effects.

However, pooling all data (across individuals and across stimuli) produced opposite results. The correlation coefficient for photographs was positive but not significant despite the comparatively high statistical power due to a larger number of cases, *r*(118) = .08, *p* = .20; one-tailed; and the correlation coefficient for outline shapes was even negative, *r*(118) = −.23.

#### Experiment 1b

**Figure 5.**
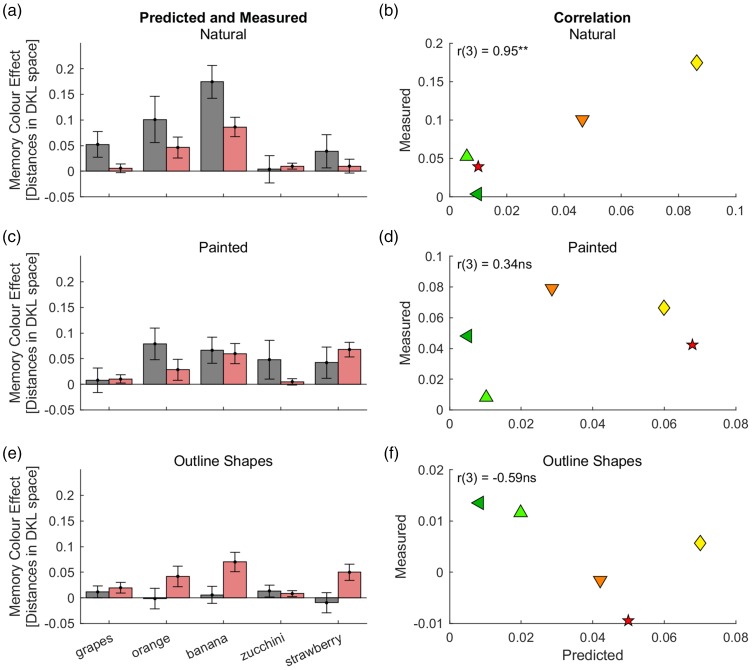
Predictions for aggregated data of Experiment 1b of Olkkonen et al. (2008). Rows illustrate results with photographs of original objects (a and b), photographs of white-painted objects (c and d) and outline shapes (e and f). Format is as shown in Figure 3. The grey bars correspond to the bars in Figure 4(b) of Olkkonen et al. (2008), except for the difference between absolute and relative shifts (cf. Figure 3).

Figure 5(a), (c) and (e) shows the results for the aggregated data of Experiment 1b. There was a tendency of the measured memory colour effects to be higher than the predicted ones for photographs, Δ_pred-meas_ = −0.04, *t*(4) = −2.8, *p* = .051, and to be lower for outline shapes, Δ_pred-meas_ = 0.03, *t*(4) = 2.5, *p* = .07.

While these tendencies just missed significance, they are in line with the observations for photographs and outline shapes in Experiment 1a and support the idea that our Bayesian model cannot account for the differences across conditions (original and painted photographs, and outline shapes). Figure 4(b) further elucidates this observation: Variance was lower for typical adjustments of outline shapes (blue bars) than for photographs of natural (green) and painted fruits (green–blue); at the same time, variances for grey adjustments were similar across conditions. This is in line with the findings from Experiment 1a (Figure 4(a)). It shows that typical adjustments of outline shapes are more reliable than those for photographs. This observation is reasonable because the adjustment of a uniform colour on the outline shape might be simpler than the adjustments of a colour distribution in the photographs. However, this makes our model predict higher memory colour effects for outline shapes, while in reality measured memory colour effects are lower. The failure of the model to predict the differences across conditions is not surprising because the model only considers knowledge about typical colours, and visual features are not part of the model.

With respect to the variation across stimuli, there was a significant positive correlation across the five fruits for photographs, *r*(3) = .95, *p* = .006, one-tailed. For the painted fruits, the correlation coefficient was positive but not significant, *r*(3) = .34, *p* = .29. However, the correlation coefficient was negative for outline shapes, *r*(3) = −.59. Combining the data from the three conditions (original and painted photographs and outline shapes) resulted in a positive correlation, which, however, did not reach significance, *r*(13) = .43, *p* = .054, one-tailed. Using the relative memory colour effects so as to compensate for the effect of condition yielded a significant positive correlation, *r*(13) = .63, *p* = .006, as found for Experiment 1a above.

However, *t* tests across individual observers did not yield significant results in any condition (all *p* > .12). There were also no significant results when pooling data across individuals and stimuli (all *p* > .1).

To summarize, the results for the aggregated data confirm those found for the other dataset above (Experiment 1a). However, the Bayesian model provides inconsistent results for the individual data. This may be due to the fact that adjustments are subject to measurement noise that is not due to sensory uncertainty. Measurement noise results, for example, from errors in handling the input device to control and confirm adjustments. This kind of noise is not accounted for in this particular model. The variability of adjustments resulting from response noise reduces the reliability of both the grey settings and the measured memory colour effects. Since this random noise should be equal across objects, adding noise to the model reduces its predictive power across objects. Hence, aggregated data might result in a more successful model because the random noise is averaged out.

In addition, the Bayesian model failed to predict the pooled data despite high statistical power due to the large number of cases. The pooled data include variability across observers. The failure to model that data might indicate that the Bayesian model fails to account for differences across individual observers. Observers considerably differ not only in their uncertainty, but also in other sources of variation that are not accounted for by the model and contribute to measurement noise. Measurement noise reduces the measured memory colour effects. In contrast, it does not have the same attenuating effects on the predictions by the Bayesian model, in particular because a noisier sensory signal would predict higher memory colour effects. In this way, individual variation may undermine the relationship between predicted and measured effects.

## Study of Witzel et al. (2011)

Witzel et al. (2011) measured memory colour effects with man-made objects that could have a wide range of different memory colours and showed the modulation of memory colour effects along the daylight axis.

### Method

A total of 25 German observers participated in the experiment of Witzel et al. (2011). This experiment included 14 images of man-made objects that were shown to be highly colour-diagnostic for German observers in a preliminary experiment. The images were a photo of a mailbox (yellow memory colour), a photo of a glue stick (yellow), a cartoon character ‘Maus’ (orange), a chair (brown), a closet (brown), an outline shape of a heart (red), a coca-cola logo (red), a photo of a fire extinguisher (red), a cartoon character ‘Pink Panther’ (pink), a chocolate bar ‘Milka’ in typical wrapping (purple), a photo of a Nivea tin (blue), a cartoon character ‘smurf’ (blue), an icon of a traffic sign (blue) and a pingpong table (green). There were also two control stimuli that did not have a chromatic memory colour. These were a striped sock that does not have any memory colour and a golfball that has an achromatic memory colour. Observers completed three adjustments for each stimulus. Before doing those adjustments, the sock was shown in its original colour (red–orange) to enable observers to make typical adjustments.

### Results and Discussion

**Figure 6.**
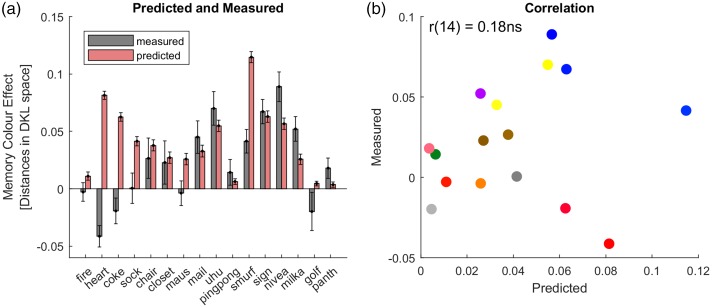
Predictions for aggregated data of Witzel et al. (2011). Panels and format are as shown in Figure 2.

Figure 6 illustrates the results for the aggregated data from Witzel et al. (2011). For many objects, the Bayesian model provided quite accurate predictions (Figure 6(a)), such as for the chair, the closet, the mailbox (‘mail’), the glue stick (‘uhu’), the pink-pong table (‘pingpong’), the traffic sign (‘sign’) and the Nivea tin (‘nivea’). Measured memory colours were highest for yellow and blue colours along the daylight locus (cf. grey bars in Figure 6(a)). This general observation was captured by the Bayesian model, in that it also predicted high memory colour indices for blue and yellow objects. At the same time, the model correctly predicted low memory colour effects for the fire extinguisher and the pingpong table whose colours do not lie along the daylight locus.

However, for some other objects the model failed. In particular, the measurements for red heart, coca-cola logo, the orange Maus and the colour-neutral sock did not yield any memory colour effects (Witzel & Hansen, 2015; Witzel et al., 2011). Nevertheless, the model incorrectly predicted high memory colour effects for these objects. For the smurf, it predicted by far the highest memory colour effect, while the measured effect was only moderate. As a consequence of these failures, there was no correlation between predicted and measured memory colour effects, *r*(14) = .18, *p* = .25, one-tailed; Figure 6(b).

It may be noted that the heart, the coca-cola logo (‘coke’) and the golf ball yielded opposite effects, that is, negative memory colour indices. By design, the model cannot predict negative effects. Furthermore, the prediction of a positive memory colour effect for the sock is based on typical measurements of short-term memorization of the sock’s colour. Yet, memory colour effects require life-long experience and cannot be produced with knowledge acquired through short-term training (Witzel & Hansen, 2015). The model, however, cannot make a difference between life-long experience and short-term training. We recalculated the correlation when excluding heart, coke, sock and golf ball because the model cannot be expected to predict the measured effects for those objects. In this case, the correlation was just significant in a one-tailed test, *r*(10) = .55, *p* = .03. For explorative purposes, we also calculated the correlation after excluding the smurf. This correlation would have been significant, *r*(9) = .82, *p* = .001.

Average correlation coefficients for the individual data of the 25 participants were not above zero (Mr = −0.04). There was not even a positive correlation when excluding data for heart, coke, sock and smurf (Mr = −0.1). Moreover, the correlation across individual observers and stimuli was not positive either, *r*(398) = −.04, even when excluding heart, coke, sock and smurf (Mr = −0.18). According to these results, our simple Bayesian model cannot predict memory colour effects across objects.

In sum, the Bayesian model could not account for the memory colour effects found by Witzel et al. (2011). This was the case despite the apparent relationship between the size of memory colour effect and the uncertainty of grey settings along the daylight locus, which should have led to a successful application of the Bayesian model. One reason for the failure of our Bayesian model for this dataset is certainly that this particular model cannot predict negative shifts, that is, shifts in the direction of the typical colour. Red objects resulted in negative shifts that contradict memory colour effects. It is not clear why red objects do not produce reliable memory colour effects (Witzel & Hansen, 2015; Witzel et al., 2011). In any case, our results show that the Bayesian model in its current implementation cannot account for this particularity of red objects (Figure 6(b)).

Another reason for the failure of our model for the data of Witzel et al. (2011) may be the mixture of photographs (e.g., Milka, Nivea and mailbox) and uniformly coloured shapes (e.g., heart, smurf, Maus and pink panther). The results for the data of Olkkonen et al. (2008) showed that the Bayesian model could not account for differences between photographs and outline shapes. Like for outline shapes, the model overestimated the magnitude of the memory colour effect for uniformly coloured shapes, such as the heart, the smurf and the Maus, which are basically outline shapes, some of them with black contours. So, perhaps the failure of this Bayesian model to predict memory colour effects for these objects has similar reasons as the failure to predict the difference in memory colour effects for photos and outline shapes.

## Conclusion

This study investigated a simple Bayesian model of memory colour effects that would link memory colour effects to more general effects of cue integration (Witzel & Gegenfurtner, in press). We reanalysed the data of three studies (Hansen et al., 2006; Olkkonen et al., 2008; Witzel et al., 2011) to test whether our Bayesian model can accurately predict memory colour effects and in particular their variation across stimuli. Our results were mixed.

On the one hand, the model predicted memory colour effects for the photographs of fruits in Hansen et al. (2006) and Olkkonen et al. (2008) very well for aggregated data. When accounting for the difference between photographs and outline shapes, the model also succeeded to predict, more generally, variations of memory colour effects across fruit stimuli.

On the other hand, the model failed to predict the null effects for certain stimuli, such as the coca cola logo, the outline-shape of a heart and the cartoon-image of a smurf. The model could also not account for the different magnitudes of memory colour effects for original photographs, photographs of white-painted fruits and outline shapes. Most importantly, by definition, Bayesian models are meant to account for data at the individual level by modelling uncertainty in each individual observer. However, while our Bayesian model shows some success in predicting aggregated memory colour effects by modelling uncertainty across observers, it failed to predict memory colour effects for individual observers.

One important reason for the failures of our model is likely to be its restriction to uncertainty about perceived grey and memorized colours. Other sources of variability, such as limited discriminability or response noise due to difficulties with the adjustment technique, are not integrated into this model. Integrating these sources of variability would require not only an extension of the Bayesian model but also additional measurements of discriminability and response noise independent of memory colour effects. Another challenge for a complete model of memory colour effects is the integration of noncolour-related factors, such as recognizability.

Nevertheless, it should be noted that the great advantage of our Bayesian model is its simplicity. It is completely determined by empirical data and does not have any free parameters. Despite its simplicity, this model succeeded in predicting the magnitude of memory colour effects for photographs in the aggregate, and also captured some of the variation of effects across stimuli. In addition, the source of the variation in memory colour effects across the three image types (i.e., different kinds of photos and outline shapes) is most probably their recognizability rather than the uncertainty about colour knowledge. It is in line with the logic of the design of our Bayesian model that it only accounts for variation in colour knowledge but not for variation of recognizability.

Taken together, our Bayesian model provides a particularly simple, yet only partial account of memory colour effects. It captures some of the multiple sources of variation of these effects and misses others. These findings provide a starting point for developing more elaborate models that include other sources of variation to fully account for differences of memory colour effects across stimuli and observers. In particular, our simple Bayesian model may be used as a benchmark to assess new alternative models.
